# Predator‐specific mortality of sage‐grouse nests based on predator DNA on eggshells

**DOI:** 10.1002/ece3.70213

**Published:** 2024-10-28

**Authors:** Nolan A. Helmstetter, Courtney J. Conway, Shane Roberts, Jennifer R. Adams, Paul D. Makela, Lisette P. Waits

**Affiliations:** ^1^ Idaho Cooperative Fish and Wildlife Research Unit, Department of Fish and Wildlife Sciences University of Idaho Moscow Idaho USA; ^2^ U.S. Geological Survey, Idaho Cooperative Fish and Wildlife Research Unit University of Idaho Moscow Idaho USA; ^3^ Idaho Department of Fish and Game Boise Idaho USA; ^4^ Department of Fish and Wildlife Sciences University of Idaho Moscow Idaho USA; ^5^ U.S. Bureau of Land Management Boise Idaho USA

**Keywords:** cattle, *Centrocercus urophasianus*, corvid, coyote, grazing, Idaho, nest survival

## Abstract

Greater sage‐grouse (hereafter sage‐grouse; *Centrocercus urophasianus*) populations have declined across their range. Increased nest predation as a result of anthropogenic land use is one mechanism proposed to explain these declines. However, sage‐grouse contend with a diverse suite of nest predators that vary in functional traits (e.g., search tactics or hunting mode) and abundance. Consequently, generalizing about factors influencing nest fate is challenging. Identifying the explicit predator species responsible for nest predation events is, therefore, critical to understanding causal mechanisms linking land use to patterns of sage‐grouse nest success. Cattle grazing is often assumed to adversely affect sage‐grouse recruitment by reducing grass height (and hence cover), thereby facilitating nest detection by predators. However, recent evidence found little support for the hypothesized effect of grazing on nest fate at the pasture scale. Rather, nest success appears to be similar on pastures grazed at varying intensities. One possible explanation for the lack of observed effect involves a localized response by one or more nest predators. The presence of cattle may cause a temporary reduction in predator density and/or use within a pasture (the cattle avoidance hypothesis). The cattle avoidance hypothesis predicts a decreased probability of at least one sage‐grouse nest predator predating sage‐grouse nests in pastures with livestock relative to pastures without livestock present during the nesting season. To test the cattle avoidance hypothesis, we collected predator DNA from eggshells from predated nests and used genetic methods to identify the sage‐grouse nest predator(s) responsible for the predation event. We evaluated the influence of habitat and grazing on predator‐specific nest predation. We evaluated the efficacy of our genetic method by deploying artificial nests with trail cameras and compared the results of our genetic method to the species captured via trail camera. Our molecular methods identified at least one nest predator captured predating artificial nests via trail camera for 33 of 35 (94%) artificial nests. We detected nest predators via our molecular analysis at 76 of 114 (67%) predated sage‐grouse nests. The primary predators detected at sage‐grouse nests were coyotes (*Canis latrans*) and corvids (*Corvidea*). Grazing did not influence the probability of nest predation by either coyotes or corvids. Sagebrush canopy cover was negatively associated with the probability a coyote predated a nest, distance to water was positively associated with the probability a corvid predated a nest, and average minimum temperature was negatively associated with the probability that either a coyote or a corvid predated a nest. Our study provides a framework for implementing an effective, non‐invasive method for identifying sage‐grouse nest predators that can be used to better understand how management actions at local and regional scales may impact an important component of sage‐grouse recruitment.

## Graphical abstract

We used DNA collected from eggshells from predated sage‐grouse nests to identify nest predators. We evaluated the influence of habitat and grazing on predator‐specific nest predation. We evaluated the efficacy of our genetic method by deploying artificial nests with trail cameras and compared the results of our genetic method to the species captured via trail camera.

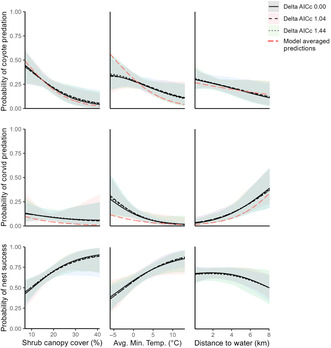

## 1 INTRODUCTION

Predation is a ubiquitous ecological process that can structure ecosystems and regulate prey populations via top‐down effects across trophic levels (Estes et al., 2011; Ripple et al., 2014). Most species contend with a diverse suite of predators that vary in density, functional traits, and habitat domain (Lima, 2002; Schmitz, 2017; Sih et al., 1998). Diversity in these traits among sympatric predators can cause variation in the consumptive and non‐consumptive effects of the predator guild on prey populations (Miller et al., 2014; Schmitz, 2017; Wirsing et al., 2021). In a sense, prey are trying to stay ahead of the proverbial predator–prey arms race (Abrams, 1986; Dawkins & Krebs, 1979) and multiple predators influence that coevolutionary relationship (Abrams, 1991) whereby trait diversity among predators may stymie the prey's ability to adapt and reduce predation risk. Additionally, ecological gradients (e.g., vegetation structure) and disturbance (e.g., anthropogenic land use) can affect predator–prey interactions by influencing predator composition and abundance, encounter rates, availability of refugia, and spatiotemporal patterns of space use by both predator and prey (Hradsky et al., 2017; Kurki et al., 1998; Lyons et al., 2015). Thus, we need to identify the explicit suite of predators in each system and the proportion of predation events attributed to each predator so that predator‐specific patterns of predation and the functional role specific predators occupy within ecological communities can be better quantified.

Identifying the predator responsible for discrete predation events (i.e., predator‐specific mortality) is a critical step in elucidating the explicit mechanisms by which ecological gradients and disturbance influence predator–prey interactions. For example, road density was the best predictor of wolf (*Canis lupus*) and bear (*Ursus* sp.) predation on mountain caribou (*Rangifer tarandus caribou*), whereas forest successional stage better predicted cougar (*Puma concolor*) predation (Apps et al., 2013). Additionally, determining predator‐specific mortality can also elucidate how the effects of predation on population dynamics can vary among predators. For example, ursid predation on neonatal elk (*Cervus canadensis*) was additive in multi‐predator systems, whereas predation of neonates by other predators (e.g., canids and felids) was partially compensatory (Griffin et al., 2011). Thus, determining predator‐specific morality and employing predator‐specific analyses to better understand predator–prey relationships enhances our ability to make well‐informed management decisions regarding prey populations and the relative importance of predation in prey population dynamics (Forrester & Wittmer, 2019; Griffin et al., 2011; Hradsky et al., 2017; Kunkel & Pletscher, 1999).

Nest predation is the primary cause of nest failure for many birds and, hence, can strongly influence habitat selection, life‐history evolution, and population growth (Conway & Martin, 2000b; Dillon & Conway, 2018; Martin, 1993; Ricklefs, 1969; Taylor et al., 2012). Avian nest predator guilds are diverse and can include many species of mammals, reptiles, and other birds (Coates et al., 2008; Kirkpatrick & Conway, 2010; Newton, 1998; Pietz & Granfors, 2000). As a result, the effects of habitat attributes (e.g., vegetation structure and composition) on nest survival can vary across species, and also over ecological gradients in conjunction with predator composition and abundance (Benson et al., 2010; Coates & Delehanty, 2010; Cox et al., 2012; Lyons et al., 2015). Determining predator‐specific nest mortality is rare in avian nest predation studies despite the critical insights it can provide into causes of nest failure, and knowledge of a species' explicit nest predators can aid in local and regional efforts to increase nest success and abundance of birds (Lahti, 2009; Thompson III, 2007; Thompson III & Ribic, 2012).

Cameras placed at nest sites have been used to document predator‐specific nest mortality in birds (Kirkpatrick & Conway, 2010; Larivière, 1999; Richardson et al., 2009). However, deploying and maintaining cameras can disturb nest sites and increase the risk of nest abandonment and detection of nests by predators via visual and olfactory cues (Larivière, 1999, Pietz & Granfors, 2000, Renfrew & Ribic, 2003, Williams & Wood, 2002; but see Richardson et al., 2009). As a result, using cameras to document patterns of nest predation carries added risks of negatively affecting the ecological variable (i.e., nest fate) under study. Further, investigators risk missing predation events that occur prior to camera deployment or misallocating resources by deploying cameras at successful nests. Recent advancements in molecular techniques have provided powerful tools for assessing predator‐specific mortality (Onorato et al., 2006; Williams et al., 2003). Predator DNA can be collected at kill sites from saliva, hair, and scat, and used to infer the species of predator responsible for each mortality event (Caniglia et al., 2013; Mumma et al., 2014; Onorato et al., 2006). Yet, only a handful of studies have utilized these noninvasive techniques for determining the predator responsible for avian nest predation events (i.e., predator‐specific nest predation; Hopken et al., 2016; Innes et al., 2015; Steffens et al., 2012). Building upon non‐invasive methods for determining predator‐specific nest predation (e.g., collecting predator DNA from predated nests) would therefore alleviate the negative effects of nest cameras while also providing valuable information regarding predator‐specific patterns of nest predation.

Greater sage‐grouse (hereafter sage‐grouse; *Centrocercus urophasianus*) populations have declined across their range (Coates et al., 2022) and declines have been attributed to land use, habitat loss, and habitat degradation (Beck & Mitchell, 2000; Kirol et al., 2015; LeBeau et al., 2014; Sandford et al., 2017). Increased nest predation as a result of land use activities is one common mechanism proposed to explain sage‐grouse population declines (Beck & Mitchell, 2000; Knick & Connelly, 2011; Webb et al., 2012). Nest predation is the primary cause of sage‐grouse nest failure and population growth is sensitive to changes in nest success (Moynahan et al., 2007; Taylor et al., 2012). Thus, like many ground‐nesting birds, changes in nest predator composition, predator abundance, or nest encounter rates could impact sage‐grouse population dynamics (Evans, 2004). Cameras have been used to identify common sage‐grouse nest predators and the habitat characteristics associated with nest success can vary in relation to predator species (Coates & Delehanty, 2010). However, a significant knowledge gap still exists regarding how heterogeneity in habitat and land use influence predator‐specific probabilities of sage‐grouse nest predation despite nest fate playing a key role in juvenile recruitment and sage‐grouse population dynamics (Coates et al., 2016; Coates & Delehanty, 2010; Moynahan et al., 2007; Taylor et al., 2012).

Domestic cattle (*Bos taurus*) grazing (hereafter grazing) is the primary land use activity across much of the sagebrush biome in western North America (Veblen et al., 2014). Grazing is often assumed to adversely affect sage‐grouse recruitment by reducing residual grass height (and hence cover), thereby increasing nest predation risk by facilitating olfactory and visual detection of nests by predators (Beck & Mitchell, 2000). The nest concealment hypothesis is commonly proposed to explain variations in nest fate in birds (Borgmann & Conway, 2015). For sage‐grouse, the nest concealment hypothesis hinges on evidence that grass height influences sage‐grouse nest success (Doherty et al., 2014) and predicts that both grass height at sage‐grouse nests and average nest success will be lower in actively grazed areas (hereafter pastures) compared to non‐grazed pastures. While grass height at both random points and nest sites is indeed lower on pastures grazed at higher intensities (i.e., pastures with more cattle per acre and/or increased grazing durations), recent evidence found little support for the hypothesized effects of grazing on nest fate at the pasture scale (Smith et al., 2018). No differences in sage‐grouse nest success among pastures grazed at different intensities suggests that either grazing fails to induce the increased nest detection by predators as commonly predicted by the nest concealment hypothesis, or grazing does induce increased nest detection, but that increase is offset by localized responses of one or more nest predators (i.e., a reduction in predator density or relative use within pastures while cattle are present). Hence, identifying predator‐specific causes of nest failure is critical to understanding causal mechanisms linking grazing to patterns of sage‐grouse nest success, so that land managers can balance grazing activities with sage‐grouse conservation.

A reduction in prey density or relative use within a pasture by predators due to increased human activity could explain why sage‐grouse nest success does not differ among pastures grazed at different intensities despite the reduction in grass height on more heavily grazed pastures (Smith et al., 2018). Mammalian carnivores will often partition themselves in space and (or) time to avoid human‐associated activities (Moll et al., 2018; Suraci et al., 2019; Van Dyke et al., 1986). While several mechanisms can drive these shifts in predator foraging activity (e.g., fear, persecution, hunting, or changes in prey abundance), a local reduction in the number of predators or changes in the relative use of areas could reduce the consumptive and non‐consumptive effects of predation on prey populations. The cattle avoidance hypothesis predicts a decreased probability of at least one sage‐grouse nest predator predating sage‐grouse nests in pastures with livestock relative to pastures without livestock present during the nesting season. Under the cattle avoidance hypothesis, increased nest detection due to reduced cover could still occur (i.e., the cattle avoidance hypothesis is not an alternative to, or mutually exclusive of, the nest concealment hypothesis). However, reduced density or within pasture use by one or more sage‐grouse nest predator(s) in response to the presence of cattle could dampen the effects of increased nest detection by reducing the number of predators foraging within pastures with active grazing. That is, the effects of the two mechanisms would counteract each other if both were valid.

Our objectives were: (1) evaluate the efficacy of using a non‐invasive molecular technique to identify sage‐grouse nest predators, and (2) test whether the cattle avoidance hypothesis counteracts the reduced concealment in grazed pastures by testing explicit predictions regarding how livestock grazing, habitat, and nest‐site characteristics influence predator‐specific nest predation. We tested the following prediction of the cattle avoidance hypothesis: the probability of mammalian nest predation on sage‐grouse nests will be lower on pastures with concurrent livestock grazing. Additionally, we tested whether (1) increased nest concealment (e.g., sagebrush canopy cover and visual concealment at nest sites) reduced the probability of avian nest predation, (2) landscape features associated with mammalian predator movement and space use increased the probability of mammalian nest predation, and (3) landscape characteristics that facilitate nesting and perching of avian nest predators or offer potential subsidies increased the probability of avian nest predation.

## 2 MATERIALS AND METHODS

### 2.1 Study area

**FIGURE 1:**
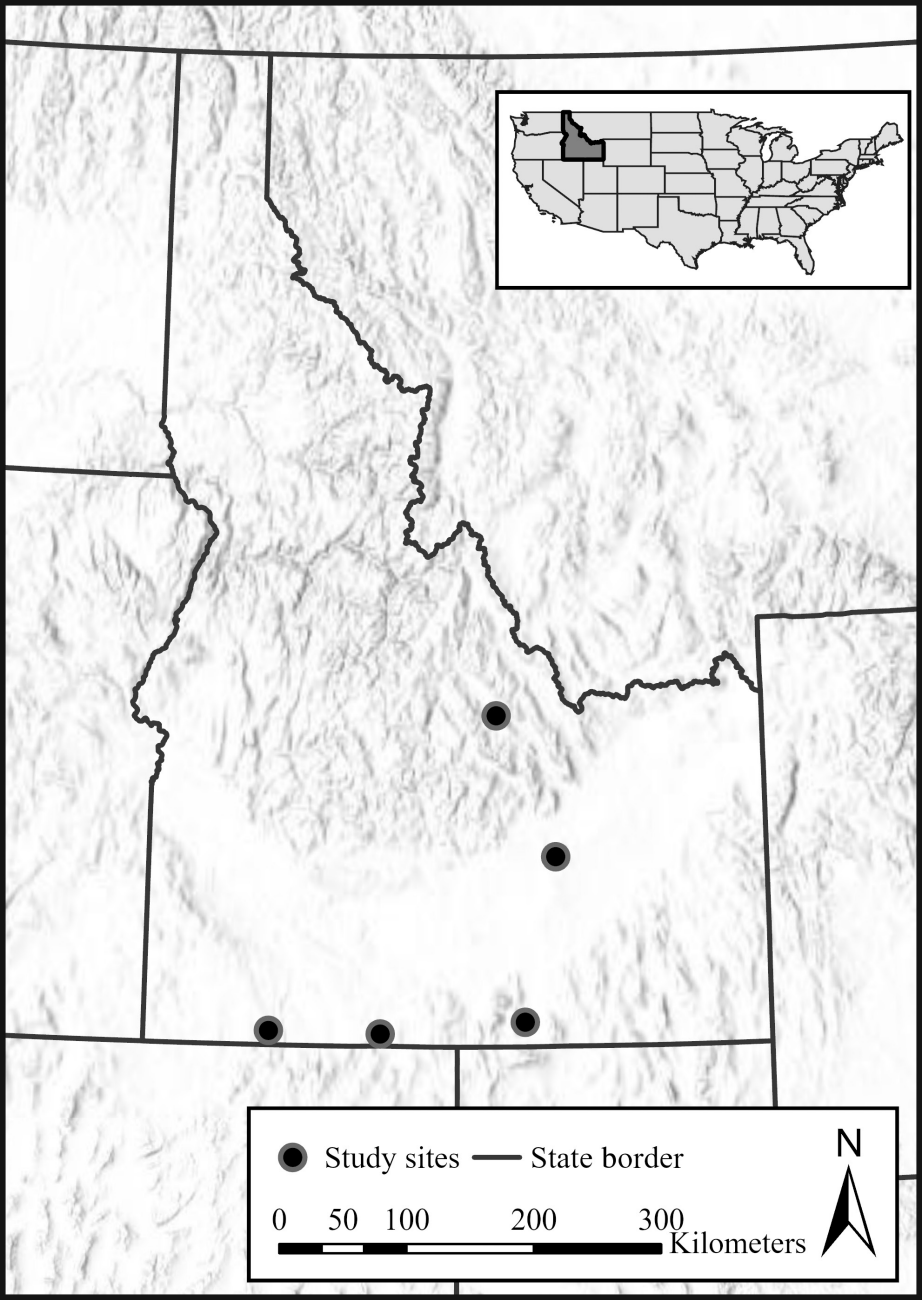
The locations of our five study sites (black circles) in Idaho, USA.

We conducted research across five study sites in southern Idaho (Figure 1; Conway et al., 2019). Elevations at the study sites ranged from 1400 to 1900 m. Common overstory plants included Wyoming big sagebrush (*Artemisia tridentata wyomingensis*), little sagebrush (*Artemisia arbuscula*), three‐tip sagebrush (*Artemisia tripartita*), rubber rabbitbrush (*Ericameria nauseosa*), and green rabbitbrush (*Chrysothamnus viscidiflorus*) (Conway et al., 2019). Sandberg bluegrass (*Poa secunda*), bottlebrush squirreltail (*Elymus elymoides*), bluebunch wheatgrass (*Pseudoroegneria spicata*), western wheatgrass (*Pascopyrum smithii*), and needle grass (*Acnatherum* spp. and *Hesperostipa* spp.) were common understory plants (Conway et al., 2019). The study sites were remote with little development in the surrounding area and were actively used as nesting habitats by sage‐grouse hens (Conway et al., 2019). Common sage‐grouse predators at our study sites included coyote (*Canis latrans*), American badger (*Taxidea taxus*), red fox (*Vulpes vulpes*), bobcat (*Lynx rufus*), raven (*Corvus corax*), crow (*Corvus brachyrhynchos*), and magpie (*Pica hudsonia*). The study sites were managed by the Bureau of Land Management (BLM) and consisted of a patchwork of fenced pastures designed to keep domestic cattle in (or out) while remaining permeable to wildlife. The BLM permits grazing to qualified applicants in a manner consistent with the Idaho Standards for Rangeland Health and Guidelines for Livestock Grazing Management (Bureau of Land Management, 1997) and the Grazing Administration Regulations (43 CFR 4100, 2005). Each permit or lease issued by the BLM specifies the type and number of livestock, the period(s) of use, the amount of use (specified in animal unit months; AUMs), and shall not exceed the livestock carrying capacity of the allotment. The Standards, relative to sage‐grouse habitat objectives, are informed by the Bureau of Land Management (2015) and Stiver et al. (2015).

### 2.2 Monitoring sage‐grouse nests

We used spotlights and hand nets to capture sage‐grouse hens at night (Conway et al., 2019; Wakkinen et al., 1992) in February and March of 2020 and 2021. We fitted sage‐grouse hens with a necklace‐type VHF radio‐transmitter (Advanced Telemetry Systems, Isanti, MN) and attempted to monitor radio‐marked hens every 2–3 days. We minimized the number of times we were within 100 m of a nest and tried to never flush a hen off her nest. We triangulated each hen's location and identified the potential shrub or cluster of shrubs that the hen was likely nesting under. We used binoculars to locate and confirm nests visually when possible. We established monitoring points 90° to 150° apart and took bearings to nesting hen locations. We considered hens to be incubating a nest if the bearings were consistent for 2–3 monitoring occasions. We carried out visual inspections of nests when hens were away from their nests during a monitoring occasion to determine whether nests had been predated. We fitted a subset of hens at one study site with platform transmitting terminal (PTT; Microwave Telemetry Inc., Columbia, MD) backpacks and we monitored those hens via GPS locations downloaded remotely. We located nests in‐person and determined nest fate when the GPS locations of a hen were inconsistent with nesting behavior and movement.

### 2.3 DNA sample collection from predated sage‐grouse nests

We collected predator DNA from eggshells at predated nests by either swabbing eggs and eggshell fragments or collecting eggshell fragments. We wore gloves to avoid contaminating samples. We dipped swabs (foam swab with polystyrene handle, 25–1506 1PF 100, Puritan Medical Products) into 1× phosphate‐buffered saline (PBS; pH 7.0, 1.37 M NaCl, 27 mM KCl, 100 mM Na_2_HPO_4_, 18 mM KH_2_PO_4_) prior to swabbing the eggshells. We swabbed the entire surface area (exterior and interior) of any eggshell fragment larger than 1/8 of an entire egg. If eggs were nearly whole (e.g., if an avian nest predator pecked a hole in the egg to remove the contents without breaking the egg apart), we only swabbed the exterior of the egg and concentrated swabbing around the edges of the hole. We used one swab per egg. When we could only locate eggshell fragments, we estimated how many fragments made up an entire egg and used one swab per the group of fragments we estimated as one egg. We broke the handle off the swabs and stored swabs in 2‐mL screw‐top tubes with 0.7‐mL of Queens Lysis Buffer (0.1 M Tris (pH 8.0), 0.1 M EDTA (pH 8.0), 0.01 M NaCl, 0.5% SDS). We collected any eggshell fragments that were <1/8 of an entire egg and stored them in 15‐mL tubes (Nalgene Straight‐Side Jar, 2118–9050, Thermo Scientific) with 2 mL of Queens Lysis Buffer, ensuring that all eggshell fragments were submerged in the buffer. We collected field negatives at four of the study sites by swabbing and collecting eggshell fragments from successful sage‐grouse nests (*n* = 10 nests, 54 samples) using the methods described above.

### 2.4 Vegetation and study site surveys

We conducted vegetation surveys at successful and failed sage‐grouse nests following procedures outlined in Conway et al. (2019). Briefly, vegetation surveys included several components: (1) photographs taken of a 20‐cm pink ball placed in the nest bowl to estimate nest concealment from four sides (lateral concealment) and top down (aerial concealment), (2) two 30‐m line‐intercept transects to estimate percent shrub canopy cover, average grass height, average of grass removed (i.e., grazing intensity), and average effective cover of vegetation, (3) estimates of percent vegetation cover from Daubenmire plots, and (4) a count of new and old wildlife and domestic livestock fecal droppings along the 30‐m line‐intercept transects (Conway et al., 2019). Additionally, we surveyed study sites and recorded locations of perches that could be utilized by avian predators as well as ephemeral and perennial water sources.

### 2.5 Proof‐of‐concept study: Artificial nests

We deployed artificial nests with motion‐sensor trail cameras (Cuddeback 8mp and 20mp White Series and Reconyx Hyperfire models) to compare the accuracy of our molecular results to the predator species captured via trail cameras. We deployed artificial nests at four of our study sites. We placed 4–5 white or brown domestic chicken (*Gallus gallus domesticus*) eggs within sagebrush shrubs to loosely mimic sage‐grouse nests. Our goal was to collect DNA samples from predated nests and, thus, our artificial nests and cameras were relatively conspicuous except for when we targeted mammalian predators. We deployed olfactory lures at each nest consisting of either commercially available predator lure (e.g., Dunlap's Predator Bait) and/or carnivore urine (*Vulpes* spp. or coyote urine) placed on an absorbent cloth and hung from the shrub branches. Additionally, we placed visual lures (e.g., feathers hung from the shrub branches) at some artificial nests. We monitored artificial nests every 1–2 days. We secured trail cameras to t‐posts and aimed the camera at the “entrance” of the artificial nests (i.e., a small gap in shrub cover that a sage‐grouse could enter and exit through). We set trail cameras to take a burst of five photos continuously (i.e., no delay between bursts) when the sensor was triggered by motion. Once we discovered that an artificial nest was predated, we immediately collected DNA from eggshells using the methods described above. We also used two additional storage methods when enough eggshell remains were available: (1) we stored swab(s) in a 15‐mL tube with a desiccant capsule (Dri‐Capsules, SGC‐50, Isohelix) separated from the swab by a chemical wipe, and (2) we stored eggshell fragments in a 50‐mL tube with 5‐mL of desiccant beads (Silica Gel, S161‐212, Fisher Chemical) separated from the eggshells by a chemical wipe.

We used logistic regression with a logit link and binomial distribution to evaluate whether detection (i.e., a binary response variable representing non‐detections and detections via our molecular analysis) was influenced by the storage method. We evaluated the influence of storage method on detection by sample (and not by nest) because multiple storage methods were used per artificial nest. Cost for materials per sample varied among storage methods. Eggshells stored in 50‐mL tubes with desiccant beads and swabs stored in 2‐mL tubes with 0.7‐mL of buffer were the cheapest storage methods, with the latter being slightly less than double the cost of the former. Eggshells stored in 15‐mL tubes with 2‐mL of Queen's Lysis Buffer and swabs stored in 15‐mL tubes with a desiccant capsule were roughly five and ten times more expensive, respectively, than eggshells stored in 50‐mL tubes with desiccant beads. However, cost per sample does not include laboratory time or laboratory materials (i.e., DNA extraction; described below) which can vary based on the amount of Queens Lysis Buffer used in the storage and extraction process. We redeployed new eggs when an artificial nest was predated (or left non‐predated eggs in the nest) and considered it a new sample in our analysis (described below).

### 2.6 DNA extraction and species identification

We extracted, amplified, and analyzed all DNA samples collected from both artificial and real sage‐grouse nests at the Laboratory for Ecological, Evolutionary, and Conservation Genetics at the University of Idaho in a laboratory dedicated to processing low‐quality DNA samples. DNA was extracted from samples using the Qiagen DNeasy Blood and Tissue Kit. Protocol modifications were made for each sample type and can be found in Table 1. All sample types were digested overnight. The swabs were carried through to the first spin step of lysate in the spin column to ensure all DNA was captured on the filter. Negative controls were used in each extraction.

**TABLE 1:** Protocol modifications to the Qiagen DNeasy Blood and Tissue Kit for each sample type collected.

Sample type	Starting material	Extraction buffer volumes	Number of spins per 600 μL^a^
Swab in QLB^b^	1–2 mL tube with 600 μL QLB^b^ and swab	40 μL proteinase K 600 μL Buffer AL 600 μL EtOH	3
Eggshells in QLB	3–2 mL tubes with 600 μL QLB^c^ per tube	40 μL proteinase K per tube 600 μL Buffer AL per tube 600 μL EtOH per tube	9
Swab with desiccant capsule	1–2 mL tube with 600 μL buffer ATL and swab	40 μL proteinase K 600 μL Buffer AL 600 μL EtOH	3
Eggshells with desiccant beads	3–2 mL tubes 600 μL QLB^d^ per tube	40 μL proteinase K per tube 600 μL Buffer AL per tube 600 μL EtOH per tube	9

^a^ Number of spins needed to spin the entire volume of lysate through one spin column.

^b^ Queens lysis buffer.

^c^ Queens lysis buffer that the sample was stored in.

^d^ Queens lysis buffer used to rinse cells from eggshells.

We conducted two independent species ID multiplex PCRs and separate fragment analyses on every sample: (1) a newly developed corvid mtDNA fragment analysis designed to distinguish magpies from ravens/crows (i.e., we could not distinguish between ravens and crows; *Corvus corax* and *C. brachyrhynchos*), and (2) a mammalian fragment analysis that can identify 16 wild mammalian carnivores including potential sage‐grouse nest predators at our study sites (coyote, bobcat, and red fox; Davidson et al., 2014, De Barba et al., 2014). Both methods consist of the co‐amplification and fragment analysis of segments of the mtDNA control region (canid, ursid, and mustelid) or the cytochrome b region (felid and corvid) using dye‐labeled primers. The corvid analysis used one dye‐labeled forward primer Corvid F.

5′‐TTCTCAGCAATCCCATACATT‐3′ and two reverse primers CcoraxBrach R.

5′‐GGGCGTGAAATTTTCTGGG‐3′ and Phudsonia R 5′‐TGCTATAGTAGCAAGTAGGG‐3′. The mammalian predator analysis used two dye‐labeled forward primers SIDL.

5′‐TCTATTTAAACTATTCCCTGG‐3′ (Murphy et al., 2000) and FelidID F.

5′‐TACATACATGCYAACGGAGC‐3′ (Davidson et al., 2014) and four different reverse primers: H16145 5′‐GGGCACGCCATTAATCGACG‐3′ (De Barba et al., 2014; Murphy et al., 2000), H3R 5′‐CCTGAAGTAGGAACCAGATG‐3′ (Dalén et al., 2004), LRuf R 5′‐CCGAATATTTCATGTCTCTGAA‐3′ (Davidson et al., 2014), and PCon R 5′‐ATGACCGCAAATAGTAGTATGA‐3′ (Davidson et al., 2014). We used the polymerase chain reaction (PCR) to amplify DNA fragments. The PCR for the mammalian fragment analysis contained 1X QIAGEN Multiplex PCR Master Mix, 0.7× Qiagen Q Solution (Qiagen Inc.), 0.29‐μM SIDL, 0.20‐μM H16145, 0.13‐μM FelidID F, 0.10‐μM H3R, 0.03‐μM LRuf R, 0.03‐μM PCon R, and 1‐μL of DNA extract in a 7‐mL reaction volume. The thermal profile for the mammalian fragment analysis was an initial denaturation step of 95°C for 15 min followed by 35 cycles of 94°C for 30 s, 46°C for 90 s, 72°C for 1 min, 60°C for 30 min, and 4°C for 10 min. The PCR for the corvid fragment analysis contained 1× QIAGEN Multiplex PCR Master Mix, 0.7X Qiagen Q Solution (Qiagen Inc.), 0.07‐μM Corvid F, 0.10‐μM CcoraxBrach R, 0.10‐μM Phudsonia R, and 1‐μL of DNA Extract. The thermal profile for the corvid fragment analysis was an initial denaturation step of 94°C for 15 min followed by 13 cycles of 94°C for 30 s, a touchdown at 65°C for 90 s with a reduction in temperature by 0.4°C each cycle, and 72°C for 1 min followed by 37 cycles of 94°C for 30 s, 60°C for 90 s, and 72°C for 1 min and lastly 60°C for 30 min and 4°C for 10 min. We loaded PCR products onto an ABI3130xl DNA sequencer and used Genemapper software (Applied Biosystems) to score fragments using size‐specific bins for species. We used both positive and negative controls in each PCR, and all samples were amplified and genotyped in duplicate as a quality control measure.

We also collected DNA samples (tissue, blood, and feathers) from American badgers, magpies, ravens, crows, and raccoons (*Procyon lotor*) within our study region. Samples were collected from roadkill, private trappers, and incidental carcasses that we located. Samples were stored in 1.4 mL of DETS buffer (tissue), with desiccant beads (tissue from private trappers), and on Nobuto strips (blood), and extracted using a Qiagen DNeasy Blood and Tissue Kit. We used these samples as well as pre‐extracted samples of coyote, red fox, gray fox (*Urocyon cinereoargenteus*), wolf (*Canis lupus*), dog (*Canis lupus familiaris*), sage‐grouse, and weasel species (*Mustela* spp.) as positive controls in our molecular analyses and to test for co‐amplification between species (e.g., corvid primers amplifying coyote DNA). Lastly, we evaluated whether the primers in the mammalian fragment analysis would amplify a unique American badger DNA fragment length that would allow us to detect American badger DNA from predated sage‐grouse nests.

### 2.7 Molecular analyses

We amplified all DNA samples collected from artificial and sage‐grouse nests twice using both the corvid and mammalian species ID multiplexes (i.e., a total of 4 amplifications per sample). We considered any detection of a predator species across any number of replicates as a detection event (i.e., we did not require multiple detections across replicates when determining whether a DNA sample detected a species). We calculated detection and accuracy metrics by nest (i.e., combining all DNA samples collected at a nest) and by sample (i.e., individual swab or tube with eggshell fragments) for our proof‐of‐concept study. We calculated detection by nest by dividing the number of artificial nests in which at least one sample detected DNA by the total number of artificial nests for which we collected samples. We calculated detection by sample by dividing the number of samples collected from artificial nests that detected DNA by the total number of samples collected. We calculated the accuracy of detecting nest predators of artificial nests via our molecular analysis (and samples collected from artificial nests) by dividing the number of artificial nests (or samples collected from artificial nests) for which DNA results correctly detected the species captured via trail camera by the number of nests (or samples collected from artificial nests) that detected any nest predator DNA. We used two approaches when evaluating accuracy for artificial nests: (1) a less‐conservative approach where we considered nests and samples accurate if our molecular analysis detected the species captured via trail camera irrespective of whether they detected an additional species not captured via trail camera, and (2) a more‐conservative approach where we considered nests and samples inaccurate if our molecular analysis detected the species captured via trail camera as well as a different species not captured via trail camera (i.e., we assumed cameras had perfect detection). We calculated detection for cameras deployed at the artificial nests we collected genetic samples from by dividing the number of cameras that captured predation events by the total number of cameras deployed. We also calculated detection for real sage‐grouse nests by nest and by sample by dividing the number of nests (or samples) that detected DNA by the total number of predated nests that samples were collected from (or the total number of samples collected from predated nests).

DNA degradation is influenced by the amount of time DNA is in the environment, UV radiation, and moisture. Hence, we used logistic regression with a logit link and binomial distribution to evaluate whether detection was influenced by the final survey interval length (i.e., the amount of time between when a sage‐grouse nest was surveyed and considered active and when the nest was discovered predated), the number of samples collected at a nest, sum of precipitation during the final survey interval (PRISM Climate Group), average minimum, maximum, and mean temperature during the final survey interval (PRISM Climate Group), and an interaction term between average temperature during the final survey interval and precipitation. We evaluated the influence of these variables on detection by nest and by sample (but did not include the number of samples per nest as a covariate in the by‐sample analysis). We standardized all variables by subtracting the mean and dividing by the standard deviation. When 2 predictor variables were correlated (i.e., Pearson correlation coefficient of >0.70), we ran univariate logistic regression models for each correlated variable and selected the variable to include in our model based on Akaike's Information Criterion corrected for small sample size (AIC_c_; Burnham & Anderson, 2002). After removing correlated variables, we compared all subsets of our global model using the *MuMIn* package (Bartoń, 2022) in R (R Core Team, 2023) and selected our top model based on AIC_c_.

We quantified the proportion of each potential nest predator species detected at predated sage‐grouse nests via our molecular method by dividing the total number of species‐specific detections across all sampled sage‐grouse nests by the total number of predated sage‐grouse nests that detected any nest predator DNA. We also quantified the proportion of each potential nest predator species detected per DNA sample by dividing the total number of samples that detected a specific nest predator species by the total number of samples that detected any nest predator DNA. When an individual nest detected multiple species via one or more DNA samples collected from that nest, we assigned a proportion of that detection to each of the species detected. For example, if samples collected from an individual nest detected both raven/crow and coyote DNA, we assigned 0.5 to the proportion of nests that detected raven/crow and 0.5 to the proportion of nests that detected coyote. Similarly, if a single sample detected multiple species, we assigned a proportion of that detection to each species detected. For example, if a single sample detected both raven/crow and coyote DNA, we assigned 0.5 to the proportion of samples that detected raven/crow and 0.5 to the proportion of samples that detected coyote. Multiple predator DNA on an egg can reflect a scenario where a second predator scavenges remains of nest contents that were initially predated by a different predator species (but we have no way of distinguishing which predator was the initial and which was the scavenger).

### 2.8 Predator‐specific nest mortality analysis

We used multinomial logistic regression to evaluate how land use, nest‐site characteristics, and habitat influenced sage‐grouse nest fate accounting for predator species. For this analysis, we excluded sage‐grouse nests that did not have grazing and/or vegetation data associated with them. We pooled raven/crow and magpie detections due to low sample sizes of corvid detections via our molecular analysis at sage‐grouse nests. Further, we only included nests where coyote was the only mammalian species detected via our molecular analysis due to a small sample size of other mammalian nest predator detections (i.e., American badgers; *n* = 2 nests). Thus, our response variables were successful nests (i.e., nests that hatched 1 or more egg; *n* = 102 nests), failed nests in which only coyote was detected via our molecular analysis (*n* = 37 nests), and failed nests in which only a corvid species (i.e., magpie, raven/crow, or both) was detected via our molecular analysis (*n* = 12 nests). We considered 23 variables believed a priori to be related to predator‐specific patterns of nest predation (Table 2). We standardized all variables by subtracting the mean and dividing by the standard deviation. When two predictor variables were correlated (i.e., Pearson correlation coefficient of >0.70), we ran univariate multinomial logistic regression models for each correlated variable and selected the variable to include in our global model based on AIC_c_. The biological effects of environmental variables can be influenced by the scales of observation for covariate data (McGarigal et al., 2016; Wiens, 1989). We considered several spatial scales of shrub canopy cover (8‐ and 44‐km; Table 2) based on the estimated home range and breeding range size of ravens (6.6‐ and 40.5‐km, respectfully; Bruggers, 1988; Smith & Murphy, 1973) and the home range size for coyotes (37–47‐km; Hernández & Laundré, 2003). We also considered shrub canopy cover at nest sites estimated from the two 30‐m line transects and intermittent scales between the estimated raven breeding range and coyote home range sizes (i.e., 14‐ and 22‐km; Table 2). To optimize the spatial scale of shrub canopy cover, we ran univariate multinomial logistic regression models for each scale and selected the optimal scale based on AIC_c_. We reduced our candidate set of predictor variables further by running univariate multinomial logistic regression models for variables that would influence the probability of predation by a specific predator species in similar ways (e.g., distance to powerlines, perch sites, and nest sites) and selected the variable to include in our model based on AIC_c_. We used the *MuMIN* package (Bartoń, 2022) in R (R Core Team, 2023) to compare all subsets of our global model and selected our top model based on AIC_c_. We explored partial effects plots for each of the variables included in the top models (i.e., delta AIC_c_ < 2) that had 95% confidence intervals (CIs) that did not overlap 0.

**TABLE 2:** Variables considered in our suite of candidate models designed to identify the factors that influence predator‐specific mortality of sage‐grouse nests in Idaho.

Variable	Source
**Average minimum temperature during the final survey interval**	PRISM Climate Group
Average maximum temperature during the final survey interval	PRISM Climate Group
Average mean temperature during the final survey interval	PRISM Climate Group
**Average precipitation during the final survey interval**	PRISM Climate Group
**Average max height of grass**	Measured on‐site (Conway et al., 2019)
Average height of grass leaf	Measured on‐site (Conway et al., 2019)
**Average of grass amount removed (i.e., Grazed)**	Measured on‐site (Conway et al., 2019)
**Average effective height of grass**	Measured on‐site (Conway et al., 2019)
**Percent aerial concealment at the nest shrub**	Measured on‐site (Conway et al., 2019)
**Average percent horizontal concealment at the nest shrub**	Measured on‐site (Conway et al., 2019)
Shrub canopy cover at the nest site (900‐m Scale)	Measured on‐site (Conway et al., 2019)
**Shrub canopy cover (8‐**, 14‐, 22‐, and 44‐km Scales)	Rigge et al., 2021
**Days since last grazed**	Measured on‐site (Conway et al., 2019)
**Number of new cattle fecal droppings**	Measured on‐site (Conway et al., 2019)
**Number of old cattle fecal droppings**	Measured on‐site (Conway et al., 2019)
**Distance to fence**	BLM (2022), Measured on‐site
Distance to road	Idaho Roads, 2019, Measured on‐site
**Distance to perching structure**	Measured on‐site
**Distance to agriculture**	Dewitz and USGS (2021)
**Distance to perennial water source**	USGS (2017)
Distance to ephemeral water source	USGS (2017), Measured on‐site
Distance to perennial or ephemeral water source	USGS (2017), Measured on‐site
Distance to nesting structures (e.g., trees or cliffs)	USGS 2016, NLCD (2021), Measured on‐site
Distance to powerlines	HIFLD (2022), Measured on‐site

*Note*: Variables in bold font are those included in the global model after eliminating correlated variables, redundancy in functional relationships, and optimizing scales of effect.

## 3 RESULTS

### 3.1 Molecular analyses

Due to stochastic co‐amplification of sage‐grouse DNA by our raven/crow primers (i.e., the Corvid F and CcoraxBrach primers), we increased the annealing temperatures of our corvid fragment analysis to 61.8°C until the raven/crow primers no longer amplified sage‐grouse DNA. We re‐ran any samples collected from predated sage‐grouse nests that detected raven/crow with only the raven/crow primers at the increased annealing temperature described above. Additionally, we found that magpie positives (i.e., tissue samples) simultaneously amplified both the fragment length associated with magpie (279.55–280.75 base pairs) and the fragment length associated with raven/crow (324.7–326.61 base pairs). Thus, using only the raven/crow primer (as described above) allowed us to determine whether our molecular analysis was detecting only magpie DNA or both magpie and raven/crow DNA from sage‐grouse and artificial nests. Interestingly, 214 samples collected from sage‐grouse nests amplified one of two fragment lengths associated with wolf/domestic dog (365–368; De Barba et al., 2014) in the mammalian fragment analysis. We determined that the primers used in the mammalian fragment analysis were co‐amplifying sage‐grouse DNA by including sage‐grouse positives in our analyses. Thus, we were unable to distinguish wolf/domestic dog from host (sage‐grouse) DNA collected from predated sage‐grouse nests.

### 3.2 Molecular analyses—Artificial nests

We collected a total of 133 samples from 42 artificial nests (range = 1–8 samples per artificial nest). Using American badger positives, we determined that the existing primers in the mammalian fragment analysis amplified a unique fragment length of 321–327 base pairs that could be used to identify American badger. We detected raven/crow, magpie, coyote, American badger, and bobcat via our molecular analysis and raven, magpie, and coyote via our trail cameras on 37 artificial nests (Tables 3, 4). We had four predated artificial nests where the camera malfunctioned (i.e., did not take any photos of the predation event), and thus, detection for cameras deployed at artificial nests was 90%. We also collected two samples (one swab sample and one eggshell fragment sample) from a stray chicken egg found by a fence (likely moved by a predator from a nearby artificial nest). The four nests where the camera malfunctioned detected magpie for nine of the 10 samples collected and the two samples collected from the stray egg detected raven/crow. The four artificial nests where the camera malfunctioned and the stray egg were included in our detection analysis but excluded from our accuracy analysis. Our detection by nest and by sample was 95% and 73%, respectively. Our molecular analyses detected a species not captured via trail camera at eight artificial nests (Table 3), but the molecular analysis also detected the species captured via trail camera at six of those eight artificial nests (Table 3). A total of 13 samples collected from the eight artificial nests (1–4 samples per nest) detected a species not captured via trail camera, but our molecular analysis also detected the species captured via trail camera for six of the 13 samples (Table 4). Thus, our accuracy by nest and by sample when using our less‐conservative approach (i.e., considering nests and samples accurate when they successfully detected the species captured via trail camera regardless of whether they also detected a second species) was 94% and 93%, respectively. Conversely, our accuracy by nest and by sample when using our more‐conservative approach (i.e., considering nests and samples as inaccurate when they detected the species captured via trail camera as well as a different predator species) was 74% and 80%, respectively. Detection was 68% for swabs stored with desiccant capsules, 76% for swabs stored with Queens Lysis Buffer, 69% for eggshell fragments stored with Queens Lysis Buffer, and 83% for eggshell fragments stored with desiccant beads. Results from our logistic regression indicated that storage method did not influence detecting predator DNA (i.e., 95% CIs for all storage methods overlapped 0).

**TABLE 3:** Comparison of species‐specific detections from trail cameras (column 1) and our molecular analysis (row 2) for the proof‐of‐concept study summarized across 35 artificial nests that detected DNA.


Molecular analysis				
	Raven/crow	Magpie	Raven/crow & Magpie	Coyote	Other
Trail camera					
Raven	12	2	0	0	0
Magpie	1^c^	16^a,b,c,d^	0	2^a,b^	1^d^
Raven & Magpie	0	1	2	0	0
Coyote	1^e^	0	0	2^e,f^	1^f^

*Note*: Exponentiated letters indicate nests in which the species captured via trail camera was detected by our molecular analysis along with a different species that was not captured via trail camera. For example, genetic samples collected from four artificial nests predated by magpies detected magpie and other species not detected via trail camera: Two nests detected coyotes ^(a,b)^, one nest detected raven/crow ^(c)^, and one nest detected badger ^(d)^. Similarly, Genetic samples collected from two artificial nests predated by coyotes detected coyote and two other species not detected via trail camera: one nest detected raven/crow ^(e)^ and one nest detected bobcat ^(f)^.

**TABLE 4:** Comparison of species‐specific detections from trail cameras (column 1) and our molecular analysis (row 2) for the proof‐of‐concept study summarized across 86 samples that detected DNA collected from the 35 artificial nests (1–8 samples per artificial nest).


Molecular analysis				
	Raven/crow	Magpie	Raven/crow & Magpie	Coyote	Other
Trail camera					
Raven	29	2	0	0	0
Magpie	1	39^a,b,c,d^	1^c^	2^a,b^	1^d^
Raven & Magpie	3	2	2	0	0
Coyote	4^e,f^	0	0	5^e,f^	1^*^

*Note*: Exponentiated letters indicate samples in which the species captured via trail camera was detected by our molecular analysis along with a different species that was not captured via trail camera. For example, four genetic samples collected from artificial nests predated by magpies detected magpie and other species not detected via trail camera: two samples detected coyote ^(a,b)^, one sample detected raven/crow & magpie ^(c)^, and one sample detected badger ^(a)^. Similarly, two of the genetic samples collected from artificial nests predated by coyotes detected coyote and raven/crow ^(e,f)^. The asterisk (*) highlights that a single genetic sample collected from an artificial nest predated by a coyote detected only bobcat DNA.

### 3.3 Molecular analyses—Sage‐grouse nests

We collected 651 samples at 124 sage‐grouse nests from our five study sites: 594 samples from 114 predated sage‐grouse nests and 57 samples from 10 successful sage‐grouse nests that were used as field‐negatives. We detected raven/crow, magpie, coyote, red fox, American badger, and cougar via our molecular analysis from the 594 samples collected from predated sage‐grouse nests (Table 5). Predators were detected using our genetic method at 76 (67%) of the 114 predated sage‐grouse nests (excluding hatched nests) when samples were aggregated by nest. Predators were detected using our genetic method from 201 (34%) of the 594 samples collected from predated sage‐grouse nests when samples were not aggregated by nest. We detected a predator (magpie) via our molecular analysis from a single sample from only 1 of 10 hatched nests (i.e., from 1 of the 57 field negative samples). We detected multiple predator species from DNA samples collected from 18 predated sage‐grouse nests (Table 6). We collected 1–10 samples (μ = 6.90) per sage‐grouse nest from the 18 nests that our molecular analysis detected multiple nest predator species and 1–11 samples (μ = 4) per sage‐grouse nest from the 58 nests that our molecular analysis detected only a single nest predator species. At the 58 sage‐grouse nests where we detected a single nest predator, we detected predator DNA on 43% (*n* = 130) of the 303 samples collected. We detected DNA from a single predator at 91% (*n* = 53) of the 58 nests where >1 sample was collected. We detected predator DNA from only 1 sample at 45% (*n* = 26) of the 58 nests (including the 5 nests where only 1 sample was collected). At the 18 sage‐grouse nests where we detected >1 nest predator, we detected nest predator DNA from 57% (*n* = 71) of the 124 samples. Of the 18 nests where we detected >1 predator, 56% (*n* = 10) of the nests had >1 sample that detected DNA for multiple nest predators (Table 6). Lastly, on 32 occasions we were unable to collect samples from predated sage‐grouse nests because no eggshell remains were found at or around the sage‐grouse nest site.

**TABLE 5:** Number of nest predator species detected from 114 predated sage‐grouse nests and from 201 samples, along with the proportion of nests and samples where each species was detected.

Species detected	Number of nests	Proportion of nests	Number of samples	Proportion of samples
Coyote	47.33	62.28	126	62.69
Raven/Crow	20.33	26.75	46.50	23.13
Magpie	4.50	5.92	22	10.95
Badger	2	2.63	2	1.00
Bobcat	0.83	1.09	1	0.50
Red Fox	0.50	0.66	0.50	0.25
Cougar	0.50	0.66	3	1.49

*Note*: If nests or samples detected more than one nest predator species, each species was assigned a proportion of the total number of species detected. For example, if a coyote and magpie were detected at an individual nest (or individual sample), each species was assigned 0.5 for that nest (or that sample).

**TABLE 6:** Species detections by sample for the 18 sage‐grouse nests that our molecular analysis detected >1 predator species.

Nest ID	Total samples	Raven/crow	Magpie	Coyote	Bobcat	Cougar	Fox
F5209N1_20	7	4	—	4	—	—	—
F5224N2_21	5	—	5	2	—	—	—
F5232N1_21	8	3	2	—	—	—	—
F5242N1_21	8	—	3	2	—	—	—
F5437N1_20	6	1	—	1	1	—	—
FX0039N1_21	4	1	—	2	—	—	—
FX0138N1_20	10	—	—	1	1	—	—
FX0384N1_21	5	5	—	—	—	—	1
FX0431N1_21	9	1	—	5	—	—	—
FX0605N1_20	5	1	—	2	—	—	—
FX0608N1_21	4	1	—	2	—	—	—
FX0694N1_21	8	—	6	2	—	—	—
FX0697N1_20	8	3	—	4	—	—	—
FX0890N1_21	8	—	2	5	—	—	—
PAVAN1N1_20	9	2	—	1	—	—	—
SHCR4N1_20	6	2	—	—	—	3	—
FX0623N2_20	5	1	—	3	—	—	—
FX0623N2_20	9	3	—	1	—	—	—

Final survey intervals for sage‐grouse nests ranged from 1 to 10 days with an average of 3.12 days. We collected 1–11 samples per nest from predated sage‐grouse nests with an average of 5.43 samples/nest. We found no evidence that survey interval, number of samples per nest, ambient temperature, or precipitation affected the detection of DNA on eggshells of predated nests; the top detection model was the null model regardless of whether we evaluated detection by nest (Table 7) or by sample (Table 8) and the covariates included in all models with a ΔAIC_c_ of <2 had 95% CIs that overlapped 0.

**TABLE 7:** Top models (delta AIC_c_ < 2) evaluating detection of predator species via our molecular analysis by nest as a function of survey interval, temperature, precipitation, and total samples collected.

Intercept	Survey interval length	Precip.	Min. Temp.	Total samples	Precip. * min. Temp.	Df	logLik	AIC_c_	ΔAIC_c_	*w*
−0.29						1	−70.30	142.64	0.00	0.13
−0.30	0.34		0.32			3	−68.41	143.05	0.42	0.10
−0.30	0.25					2	−69.53	143.19	0.55	0.10
−0.30			0.22			2	−69.67	143.47	0.83	0.09
−0.30		−0.23				2	−69.71	143.55	0.91	0.08
−0.31	0.35	−0.25	0.32			4	−67.76	143.93	1.29	0.07
−0.30	0.25	−0.24				3	−68.92	144.08	1.45	0.06
−0.29				−0.12		2	−70.11	144.34	1.71	0.06
−0.30		−0.23	0.23			3	−69.37	144.38	1.74	0.05

*Note*: The asterisk (*) indicates an interaction term between total precipitation and average minimum temperature during the final survey interval. The null model (intercept only) was the top model.

**TABLE 8:** Top models (delta AIC_c_ < 2) evaluating detection of predator species via our molecular analysis by eggshell sample as a function of survey interval, temperature, and precipitation.

Intercept	Survey interval length	Precip.	Max. Temp.	Precip. * max. Temp.	Df	logLik	AIC_c_	ΔAIC_c_	*w*
−0.66					1	−358.30	718.61	0.00	0.22
−0.94			0.02		2	−357.78	719.59	0.99	0.14
−0.65		−0.02			2	−358.23	720.49	1.89	0.09
−0.63	−0.01				2	−358.28	720.58	1.97	0.08

*Note*: The asterisk (*) indicates an interaction term between total precipitation and average minimum temperature during the final survey interval. The null model (intercept only) was the top model.

### 3.4 Predator‐specific mortality

**FIGURE 2:**
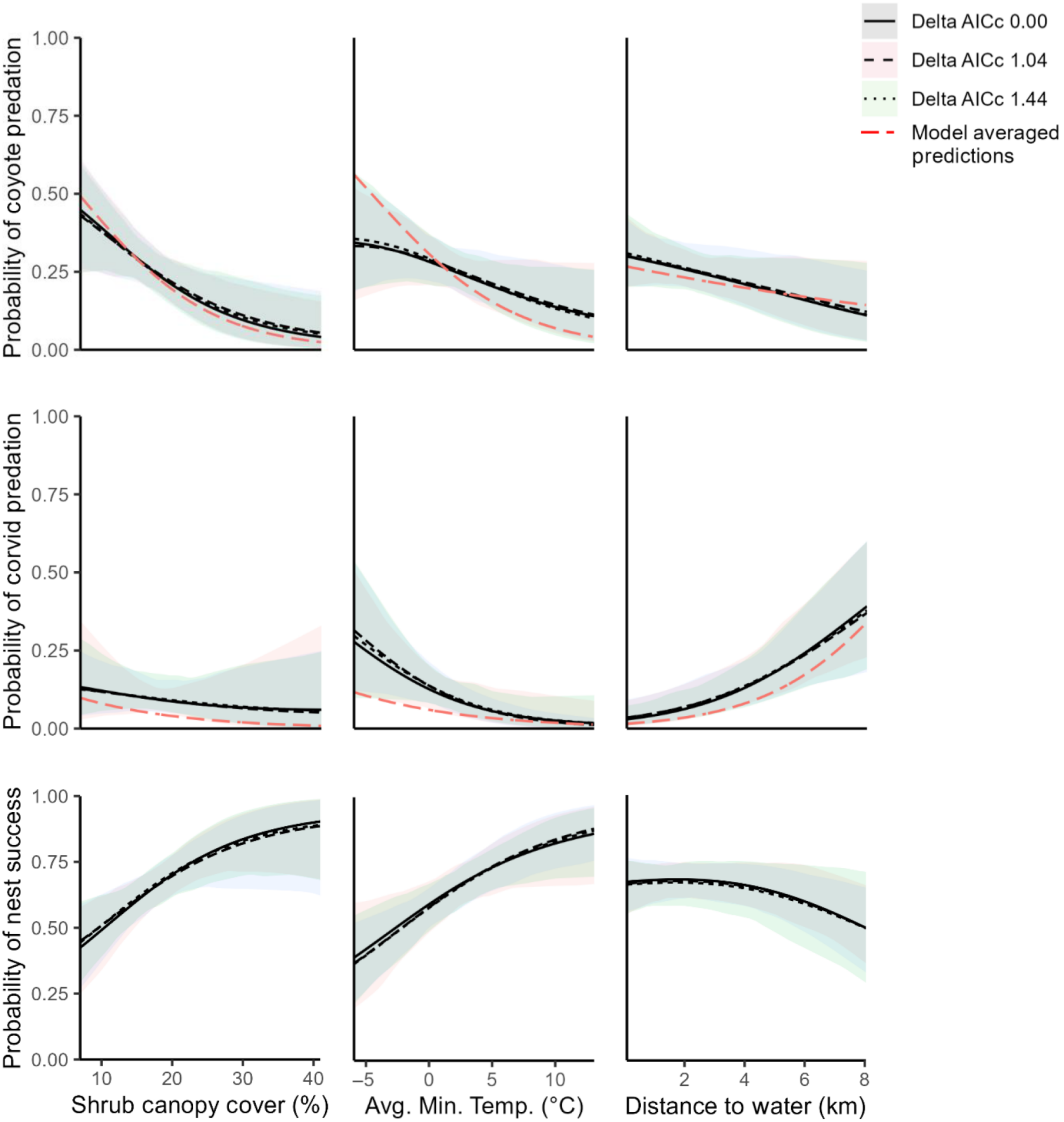
Partial effects plots for response variables in the top models evaluating factors that influence predator‐specific nest mortality. Partial effects plots are only shown for variables in the top model that had 95% confidence intervals that did not overlap 0. The black lines represent the relationship between the covariate and the response variables for the three top models with a ΔAIC_c_ < 2. The dashed red lines show the relationship between the covariates and the coyote and corvid response variables averaged across all models that encompassed 95% of the model weight.

We used 15 predictor variables in the global model after removing correlated and functionally redundant candidate variables and optimizing the scale for shrub canopy cover (bolded variables in Table 2). Shrub canopy cover influenced the probability of nest predation by coyotes, distance to a perennial water source influenced the probability of nest predation by corvids, and minimum temperature influenced the probability of nest predation by both corvids and coyotes. All other predictor variables included in the top models (Table 9) had CIs that overlapped 0. Shrub canopy cover was negatively associated with the probability that a coyote predated a nest (top model beta = −0.67, top model 95% CIs = −1.19, −0.14), distance to a perennial water source was positively associated with the probability a corvid predated a nest (top model beta = 0.91, top model 95% CIs = 0.28, 1.54), and average minimum temperature during the final survey interval prior to nest predation was negatively associated with the probability that either a coyote (top model beta = −0.46, top model 95% CIs = −0.91, −0.01) or a corvid (top model beta = −1.03, top model 95% CIs = −1.79, −0.27) predated a nest (Figure 2). Model weight was relatively low for all top models (9.0%; Table 9). Therefore, we visually compared partial effect plots of shrub canopy cover, distance to a perennial water source, and average minimum temperature to the partial effects plots of the same variables from model‐averaged estimates that encompassed 95% of the model weight to ensure that the relationships between the predictor and the probability of coyote or corvid nest predation were consistent across all models and they were (Figure 2).

**TABLE 9:** Coefficient estimates with 95% confidence intervals (CIs) for the top multinomial logistic regression models (ΔAIC_c_ < 2) evaluating factors that influence sage‐grouse nest predation by (a) coyotes and (b) corvids.

(a)										
Percent lateral cover (95% CIs)	Dist. To perch (95% CIs)	Dist. To perennial water source (95% CIs)	Precip. (95% CIs)	Percent shrub cover (95% CIs)	Avg. min. Temp. (95% CIs)	Df	logLik	AICc	ΔAIC_c_	*w*
	−0.71 (−1.49, 0.08)	−0.19 (−0.69, 0.31)		−0.67 (−1.19, ‐0.14)	−0.46 (−0.91, −0.01)	10	−103.80	229.15	0	0.04
	−0.74 (−1.55, 0.08)	−0.18 (−0.69, 0.32)	−0.53 (−1.23, 0.18)	−0.65 (−1.18, −0.12)	−0.49 (−0.93, −0.04)	12	−101.97	230.19	1.04	0.03
0.15 (−0.30, 0.60)	−0.70 (−1.48, 0.08)	−0.18 (−0.69, 0.32)		−0.70 (−1.22, −0.17)	−0.45 (−0.90, 0.00)	12	−102.20	230.60	1.44	0.02

(b)										
Percent lateral cover (95% CIs)	Dist. To perch (95% CIs)	Dist. To perennial water source (95% CIs)	Precip. (95% CIs)	Percent shrub cover (95% CIs)	Avg. min. Temp. (95% CIs)	Df	logLik	AICc	ΔAIC_c_	*w*
	−1.72 (−4.00, 0.56)	0.91 (0.28, 1.54)		−0.49 (−1.29, 0.32)	−1.03 (−1.79, −0.27)	10	−103.80	229.15	0	0.04
	−1.70 (−3.92, 0.53)	0.93 (0.29, 1.57)	0.11 (−0.47, 0.69)	−0.46 (−1.26, 0.34)	−1.07 (−1.86, −0.27)	12	−101.97	230.19	1.04	0.03
0.52 (−0.01, 1.05)	−1.78 (−4.10, 0.53)	0.95 (0.31, 1.61)		−0.44 (−1.21, 0.32)	−1.04 (−1.83, −0.25)	12	−102.20	230.60	1.44	0.02

*Note*: The table only includes covariates that were included in ≥1 of the top models (ΔAIC_c_ < 2).

## 4 DISCUSSION

### 4.1 Molecular analyses—Artificial nests

Identifying the explicit predator responsible for a predation event is a critical step in quantifying predator–prey relationships (Forrester & Wittmer, 2019; Griffin et al., 2011; Hradsky et al., 2017; Mumma et al., 2014). Our proof‐of‐concept study highlights that molecular techniques are a worthwhile method to identify both avian and mammalian nest predators from eggshell fragments at predated sage‐grouse nests. Detection of predator DNA from artificial nests was higher when evaluated by nest (i.e., pooling samples per artificial nest) than when evaluated by sample (i.e., by egg or eggshell fragments). Thus, like other studies, our proof‐of‐concept results suggest collecting multiple samples from prey remains (e.g., collecting ≥1 swab per egg for multiple eggs per nest) is an important component of increasing success rates for identifying predators via molecular techniques (Mumma et al., 2014; Sundqvist et al., 2008). Accuracy for detecting the true nest predator on artificial nests was 94% (by nest) and 93% (by sample) when evaluated using our less‐conservative approach (i.e., considering nests and samples accurate if they detected the species captured via trail camera irrespective of whether they detected an additional species not captured via trail camera) and 74% (by nest) and 80% (by sample) when evaluated using our more‐conservative approach (i.e., considering nests and samples that detected >1 predator species as inaccurate if the trail camera only detected 1 of them). Previous studies that utilized nest‐cameras to evaluate the efficacy of molecular techniques for nest predator identification had varying success. For example, Steffens et al. (2012) used general vertebrate primers to evaluate the efficacy of molecular techniques for avian and mammalian nest predator identification and reported an accuracy of 44.4%. Hopken et al. (2016) used general mammalian primers and correctly identified five of the seven (71%) mammalian predators captured via trail camera with their molecular analysis. Regardless of the method we used to evaluate accuracy (i.e., the less‐ versus more‐conservative approach), our proof‐of‐concept results indicate identifying both avian and mammalian nest predators via molecular techniques is more accurate than previously reported. Furthermore, when the predator community is known in advance, a more targeted approach (e.g., predator‐specific or taxa‐specific primers) may improve accuracy.

The accuracy of detecting nest predators via molecular techniques likely varies by species and, hence, would best be assessed separately for each species. From an applied perspective, we consider our more‐conservative approach a better representation of the efficacy of our molecular method if quantifying predator‐specific patterns of nest predation is the management objective. That is, multiple predator species may be detected via our molecular method due to scavenging events and the inability to distinguish between the initial nest‐predator and scavenger should be accounted for when considering the use of our molecular method in evaluating predator‐specific nest mortality. Lastly, when grouping avian and mammalian species into functional groups (i.e., corvids and mammals), our more‐conservative estimate of accuracy is even higher: 80% by nest and 87% by sample. Factors that influence predator‐specific patterns of nest predation (e.g., ecological gradients or land use) among functional groups are likely similar, and thus, pooling results by functional group is informative for avian conservation and management efforts.

On 4 occasions, trail cameras did not capture a species predating our artificial nests despite the cameras remaining functional the whole time. Thus, detection of nest predators via cameras was not 100% (in our case camera detection was 90%). Further, eggs were often moved from the viewshed of the camera and therefore, we were unable to determine from the camera data if an additional species scavenged the eggs after the initial predation event. We suspect that subsequent scavenging may explain why 12 samples from eight artificial nests detected DNA from species that were not captured via trail cameras. Our results indicate that camera results are not perfect (despite that assumption in our calculations of “accuracy” for our molecular methods) and both molecular methods and the use of cameras for identifying nest predators can suffer from detection and (or) accuracy issues (Coates & Delehanty, 2010; Hopken et al., 2016; Taylor et al., 2017). Thus, taking steps to increase camera detections (e.g., using higher‐quality batteries, using continuously recording cameras, or using >1 camera per artificial nest) and increasing the area that animals can be detected (e.g., include additional cameras that monitor larger viewsheds) would be helpful in future proof‐of‐concept studies.

Field collection and preservation (i.e., storage) methods of low‐quality DNA (e.g., nest predator saliva collected from predated eggshells) can influence DNA amplification success rates (Murphy et al., 2000; Roon et al., 2003; Wultsch et al., 2015). Additionally, storage methods can vary in cost and balancing the allocation of resources (i.e., time and money) is often a necessity in wildlife conservation and management. Detection of predator DNA ranged from 68–83% across our four storage methods, but we found no evidence that storage method affected the probability of detecting predator DNA from eggshells. Thus, storage methods that reduce overall cost and laboratory time may be applicable for identifying avian nest predators via our molecular method. Based on cost per sample, while accounting for laboratory time and materials, the swabs stored in buffer were the cheapest method. While eggshells stored in silica appear cheaper, increased laboratory time and materials resulted in this storage method being more costly. However, our sample sizes varied from 19 to 49 per storage method and we did not account for factors that influence DNA degradation prior to sample collection (e.g., whether there was variation in exposure to UV radiation among samples collected from individual nests). Further studies that examine the effect of storage methods that incorporate the environmental conditions that each sample was subjected to prior to collection would provide helpful insights to improve future applications of this method.

### 4.2 Molecular analyses—Sage‐grouse nests

We successfully detected the sage‐grouse nest predators responsible for nest mortality from predated sage‐grouse nests via our molecular analyses. Coyotes were the dominant nest predator among our five study sites and corvids were the second most common nest predator. One of our five sites had higher corvid densities than the other four sites (based on 3 years of point‐count survey data; C. Conway, unpublished data). Our molecular results indicated a higher proportion of corvid nest predations at the site with higher corvid densities which further supports the efficacy of our molecular technique for identifying nest predators. We detected a higher proportion of coyotes than other sage‐grouse nest predation studies have reported from Idaho and adjacent states (Coates et al., 2008; Lockyer et al., 2013; Taylor et al., 2017), and this difference may reflect one or more of the following reasons: (1) our study sites were remote with little human development and thus, may have lower corvid abundance (i.e., fewer anthropogenic subsidies for foraging and nesting; Leu et al., 2008), (2) coyotes may leave more DNA on eggshell remains compared to other mammalian and avian nest predators thus, increasing our detection rates for coyotes, or (3) past studies that relied on cameras at nests may have overestimated the proportion of corvid nest predators if the presence of a camera at a nest increases a corvid's ability to locate a nest (more so than for coyotes). We had several nests where multiple nest predators were detected and most of these “multiple” detections (*n* = 15) included an avian and mammalian species. Our molecular analyses cannot determine which species initially predated those nests, but our results suggest that scavenging eggs or eggshells from a predated nest may be a common event that is not always captured via cameras deployed at nests. Of the 18 sage‐grouse nests that detected >1 predator species, 10 of those nests detected the predators on multiple samples (i.e., DNA was detected from multiple eggs). Thus, we feel confident that our molecular analysis is indeed capturing scavenging events. Lastly, this is the first study to report evidence that a cougar either predated or scavenged a sage‐grouse nest, and we plan to confirm this result via a DNA sequence analysis.

Detection of nest predator DNA via our molecular analysis was far lower for sage‐grouse nests when evaluated by nest and sample (67% and 34%, respectively) when compared to our proof‐of‐concept study on artificial nests (95% and 73%). We unexpectedly did not find a relationship between the number of samples collected and detection rate. However, similar to our proof‐of‐concept study, detection of nest predator DNA was higher when evaluated by nest, further re‐enforcing that collecting multiple eggshell samples from predated nests increases success rates for identifying predators via molecular techniques. Lower detection of nest predator DNA from sage‐grouse nests (relative to our artificial nests) may have occurred because final survey intervals for sage‐grouse nests ranged from 1 to 10 days, whereas we collected samples from artificial nests within 2 days of a predation event (except for one artificial nest where a 1‐week gap occurred between the predation event and sample collection). However, we did not find a relationship between final survey interval length or weather on DNA detection, but both time and environmental conditions influence DNA degradation (Barnes & Turner, 2016; DeMay et al., 2013; Murphy et al., 2007). Thus, getting to samples quickly (i.e., within 1–2 days of a predation event) when collecting low‐quality DNA such as saliva on eggshells would likely increase detection. Logistically, this can be challenging and may be one potential drawback to utilizing molecular techniques to identify nest predators based on eggshell remains at sage‐grouse nests. Regardless, the number of sage‐grouse nests where we detected predator DNA (*n* = 76) is comparable to studies utilizing cameras to identify nest predators (Burr et al., 2017; Coates & Delehanty, 2010; Ellis et al., 2018; Guppy et al., 2017; Lyons et al., 2015; Staller et al., 2005), and many such studies do not explicitly report detection metrics (e.g., the number of cameras at predated nests that did not detect the nest predator) and/or camera malfunctions. Thus, results from our sage‐grouse nests, in conjunction with our proof‐of‐concept results, provide evidence that molecular techniques provide a viable alternative method for determining predator‐specific nest mortality (which can be used alone or in combination with nest cameras), and this method is especially useful for species where nest fate (and predator‐specific nest predation) may be influenced by deploying cameras at nests.

We were unable to collect samples from 32 predated sage‐grouse nests because eggshell remains were not present at the nest site. Recent studies have highlighted the ability of molecular techniques for identifying species from low‐quality environmental DNA (eDNA; e.g., identifying species from eDNA collected from soil; Leempoel et al., 2020). We suggest exploring the efficacy of using eDNA samples collected from nest sites (e.g., collecting dirt from in and around the nest bowl) as a means of identifying nest predators when eggshell remains are not present. Sampling predated nests more quickly (i.e., within 1–2 days) would also likely reduce the percentage of predated nests without eggshell fragments. Additionally, our technique was designed to target primary sage‐grouse nest predators, but other species are known to predate sage‐grouse nests (e.g., *Mephitis* spp.). Predated sage‐grouse nests in which our molecular analysis did not detect predator DNA could be the result of our inability to detect less common nest predators. Developing additional primers for the PCR multiplex that capture all potential nest predator species would enhance our technique making it transferable to areas with high abundances of less common nest predators. Similarly, investigators could leverage advances in metabarcoding techniques that would capture the full spectrum of sage‐grouse nest predators and alleviate the need to know the exact predator community in advance. In general, however, the species we were able to detect via our fragment analyses often account for most sage‐grouse nest predations throughout their range (Conover & Roberts, 2017) and thus, our analysis would still provide valuable insight into sage‐grouse nest predation dynamics. Finally, biases when using molecular analyses to identify nest predators and make inferences about predator‐specific nest mortality may be introduced for several reasons: (1) certain nest predators like snakes (*Ophidia*) may remove eggs from nests and thus, go entirely undetected, (2) certain nest predators may leave behind less DNA (e.g., avian nest predators) and thus, detection may be biased low, and (3) certain nest predator species may be prone to scavenging and thus, introduce challenges in discerning underlying mechanisms driving avian nest fate. Ultimately, the molecular results from our proof‐of‐concept study and from sage‐grouse nests indicate that investigating these potential biases to further refine this novel, non‐invasive approach for identifying nest predators is a worthwhile endeavor.

### 4.3 Predator‐specific mortality

Determining predator‐specific mortality plays a critical role in elucidating how ecological gradients, disturbance, and land use influence patterns of predation and the role specific predators play in prey population dynamics (Apps et al., 2013; Griffin et al., 2011; Lyons et al., 2015). We predicted that increased canopy cover would reduce the probability of corvid predation due to increased aerial concealment, whereas lateral concealment at a nest site would reduce the probability of mammalian predators. Our results indicate that higher shrub canopy cover decreases nest predation by coyotes (Figure 2). Coyote habitat use and movement are closely tied to prey availability and foraging success (Brunet et al., 2023; Gese et al., 1996; Moorcroft et al., 2006). Coyotes may avoid areas of high shrub canopy cover because they are a coursing predator and higher shrub canopy cover could reduce their ability to efficiently navigate the landscape in search of prey. Further, coyotes may avoid areas of higher shrub canopy cover because high shrub canopy cover reduces prey detection by reducing visibility and the permeation of smells throughout the environment, and thus, foraging success could be less efficient for coyotes in areas of high shrub canopy cover. Several studies have corroborated the positive relationship between shrub canopy cover and nest survival in sage‐grouse (Kolada et al., 2009; Lockyer et al., 2015; Webb et al., 2012), however, our study is the first to highlight a mechanistic link between shrub canopy cover and coyote‐specific nest predation risk. Current sage‐grouse management guidelines suggest that 15%–25% sagebrush canopy cover with perennial grasses and forbs in the understory is the optimal breeding habitat (Connelly et al., 2000). Yet, our results suggest that in regions where coyotes are the primary nest predator, maintaining large tracts of land with >25% sagebrush canopy cover would improve sage‐grouse nest success (Figure 2). Thus, our results emphasize the importance of accounting for predator‐specific nest mortality when making management decisions regarding sage‐grouse habitat. Shrub canopy cover was also negatively associated with the probability of corvid predation (albeit not as strong as the relationship with the probability of coyote predation; Figure 2), indicating that shrub canopy cover may be an important landscape characteristic for reducing nest predation for both avian and mammalian nest predators of sage‐grouse nests. Confidence intervals overlapped 0 for the effect of shrub canopy cover on the probability of corvid predation, but our sample size was small for nests predated by corvids. We also found a negative relationship between average minimum temperature and the probability of nest predation by both corvids and coyotes (Figure 2). Incubating hens may increase the number of recesses (i.e., foraging bouts) they take during cold weather (Conway & Martin, 2000a), and increased frequency of nest recesses can increase the detection of nests by predators (Conway & Martin, 2000b). Providing micro‐habitat features that buffer against periods of low temperatures and/or providing high‐quality forage near nests to reduce hen movement and bolster body condition during low temperatures could promote nest success in some regions. Our results also suggested a positive relationship between distance to a perennial water source and the probability of corvid predation. This positive relationship likely occurred because most of the corvid predations (58%) occurred at one study site with relatively few perennial water sources located near our surveyed nests (i.e., all surveyed nests at that study site were >3500 m from a perennial water source). We did not include the study site as a fixed factor in our models because of model convergence issues and because we were interested in factors that influence predator‐specific nest predation that were ubiquitous rather than site‐specific effects. We predicted distance to water would have a stronger and negative relationship with the probability of mammalian predation because free water sources in dry environments are frequented by mammalian carnivores and can influence carnivore distributions and space use (Abade et al., 2014; Kluever et al., 2017). As predicted, distance to perennial water source had a negative relationship with the probability of coyote predation, but CIs overlapped 0. Lastly, variables associated with grazing were not included in any of our top models and thus, our results did not support a key prediction of the cattle avoidance hypothesis (i.e., the probability of mammalian nest predation on sage‐grouse nests will be lower on pastures with concurrent livestock grazing). However, we had relatively few predated and successful nests in which cattle were present during the final survey interval or shortly thereafter (*n* = 9 and *n* = 21, respectively). Of those nine predated nests, eight detected coyote as the nest predator via our molecular analysis. Thus, we likely had too few nests to determine the effects of grazing on predator‐specific mortality. Additionally, our relatively small sample size of nests predated by corvids (*n* = 12) likely limited our ability to distinguish habitat characteristics and land use activities that influence the probability of corvid predation. The relationships were consistent among our top models and the averaged models (e.g., a negative relationship between shrub canopy cover and the probability of coyote predation), which provides confidence in the rigor of these relationships. Despite no evidence for the cattle avoidance hypothesis, our study provides a framework for implementing an effective, non‐invasive method for identifying sage‐grouse nest predators that can be used to better understand how management actions at local and regional scales may impact an important component of sage‐grouse recruitment.

Variation in the functional traits and foraging methods of predators as well as how ecological gradients and disturbance influence predator‐specific patterns of nest predation gives rise to challenges when quantifying predator–prey relationships. Nest predation can influence population dynamics for many avian species and nest predator guilds can span taxonomic classes in many predator–prey communities. Moreover, factors that influence nest fate likely vary by predator species and/or functional groups. Despite this, efforts to identify factors that influence nest fate often group predation events across predators resulting in binary analyses of failed versus successful nests (i.e., regardless of the nest predator(s) responsible). This approach risks confounding or even negating important habitat and nest‐site characteristics that may influence the probability that specific predators predate a nest. Deploying cameras at nest sites to identify the explicit predators responsible for nest predation has highlighted the spatial variation in predator‐specific patterns of nest mortality. However, deploying cameras at nests is invasive and may affect nest fate in some species or ecosystems (i.e., they may bias the explicit metric they are intended to measure). The use of non‐invasive molecular methods to identify predators of bird nests presents a novel opportunity to mitigate the challenges of both identifying the predator species responsible for nest predation events and quantifying factors that influence nest fate (which may vary among predator species) with a method that does not affect predator behavior or probability of nest detection.

## AUTHOR CONTRIBUTIONS

**Nolan A. Helmstetter:** Conceptualization (equal); data curation (lead); formal analysis (equal); investigation (equal); methodology (equal); writing – original draft (lead); writing – review and editing (equal). **Courtney J. Conway:** Conceptualization (equal); funding acquisition (equal); methodology (equal); project administration (lead); writing – review and editing (equal). **Shane Roberts:** Conceptualization (equal); investigation (equal); methodology (equal); writing – review and editing (equal). **Jennifer R. Adams:** Conceptualization (equal); methodology (equal); writing – review and editing (equal). **Paul D. Makela:** Funding acquisition (equal); resources (equal); writing – review and editing (equal). **Lisette P. Waits:** Conceptualization (equal); methodology (equal); writing – review and editing (equal).

## FUNDING INFORMATION

Funding was provided by the Bureau of Land Management, Idaho Department of Fish and Game, Idaho Governor's Office of Species Conservation, and the Public Lands Council.

## CONFLICT OF INTEREST STATEMENT

The authors declare that they have no known competing financial interests or personal relationships that could have appeared to influence the work reported in this paper.

## Data Availability

The data that support the findings of this study are openly available at https://github.com/nhelm/SageGrouseNestPredation.
